# Reoperative aortic valve replacement in the era of valve‐in‐valve procedures

**DOI:** 10.1002/ccr3.2989

**Published:** 2020-05-27

**Authors:** Daisuke Kaneyuki, Hiroyuki Watanabe, Masayoshi Otsu, Hiroaki Yamamoto

**Affiliations:** ^1^ Division of Cardiovascular Surgery Japanese Red Cross Narita Hospital Narita‐shi Japan

**Keywords:** reoperative aortic valve replacement, structural valve deterioration, transcatheter aortic valve‐in‐valve procedure

## Abstract

Current evidence suggests that the choice between valve‐in‐valve transcatheter aortic valve implantation and reoperative aortic valve replacement should be based on multiple factors.

## Graphical abstract

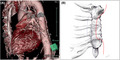

## 1 INTRODUCTION

We report a case of a 72‐year‐old man who previously underwent aortic valve replacement and coronary artery bypass grafting, presenting with acute congestive heart failure. Emergent reoperative aortic valve replacement was performed (rather than transcatheterization using a valve‐in‐valve technique) through a modified median sternotomy without injury to the bypass graft.

Reoperative aortic valve replacement (AVR) can be performed with a low risk in the present era.^1^ However, it still carries a high operative mortality risk in the emergency setting or after coronary artery bypass grafting (CABG). Transcatheter aortic valve‐in‐valve procedures are clinically effective, at least in the short term, and could be an acceptable approach in selected high‐risk patients. However, the indication for these procedures remains uncertain because of the lack of data on long‐term outcomes or poor hemodynamic outcomes.^2^ Herein, we report the case of a patient who underwent emergent reoperative AVR through a modified median sternotomy after AVR and CABG.

## 2 CASE HISTORY

A 72‐year‐old man with diabetes mellitus, chronic kidney disease, chronic hepatitis C, and a history of a brain contusion presented with acute chest pain and dyspnea. He had a history of AVR (23‐mm Mosaic bioprosthesis, Medtronic Inc) for aortic stenosis with CABG (left internal thoracic artery [LITA] to the left anterior descending artery and saphenous vein graft to the posterior descending artery) 12 years previously. His peak velocity and mean transaortic gradient had increased gradually over time.

## 3 DIFFERENTIAL DIAGNOSIS, INVESTIGATIONS, AND TREATMENT

**Figure 1:**
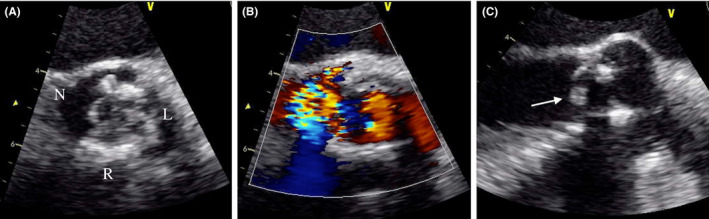
Preoperative transesophageal echocardiography. A, B, Severe aortic regurgitation at the torn noncoronary cusp (R: right coronary cusp, L: left coronary cusp, N: noncoronary cusp), and (C) a torn, prolapsed noncoronary cusp (white arrow)

**Figure 2:**
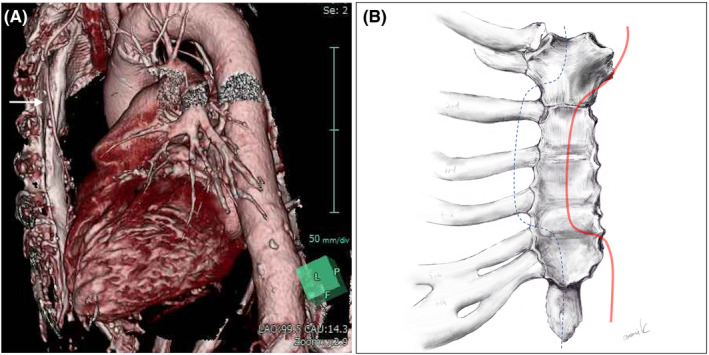
A, Preoperative three‐dimensional enhanced computed tomography revealed a patent left intrathoracic artery graft just below the sternum (white arrow); (B) depiction of modified median sternotomy with dissection of the second, third, and fourth right costal cartilages (blue line: sternotomy line, red line: left intrathoracic artery graft)

He was intubated because of severe respiratory distress. An electrocardiogram showed a negative T wave and slight ST elevation in the I, aVL, and V5‐6 leads. Although his cardiac enzymes were not significantly elevated, transthoracic echocardiography revealed decreased anterior left ventricular wall motion and a slightly abnormal left ventricular ejection fraction (44%), with the peak velocity and mean transaortic gradient increasing to 4.5 m/s and 46 mm Hg, respectively. Coronary angiography revealed a patent LITA and saphenous vein graft without new lesions in the native coronary arteries. Therefore, he was diagnosed with acute congestive heart failure due to prosthetic aortic valve stenosis. After medical treatment, his respiratory symptoms improved. However, 1 day after extubation, he suddenly developed respiratory distress again with paroxysmal atrial fibrillation. Transesophageal echocardiography after re‐intubation showed severe aortic regurgitation due to a noncoronary cusp tear (Figure 1). He was afebrile, and blood cultures on admission were negative. Acute aggravation of structural valve deterioration was suspected because of increased pressure on the calcified noncoronary cusp after extubation. Preoperative enhanced computed tomography showed that a patent LITA graft was located immediately beneath the sternum (Figure 2). Blood tests showed decreased hematocrit, platelet count, and renal function. Considering his severe congestive heart failure requiring inotropic agents and the need for emergent surgery, his Society of Thoracic Surgeons Predicted Risk of Mortality score was 28.2%. Although the transcatheter valve‐in‐valve technique was considered, there was a risk of migration of the torn noncoronary cusp. Therefore, he underwent emergent reoperative AVR.

The femoral artery and vein were exposed and taped. A modified median sternotomy was performed with an oscillating saw with second, third, and fourth costal cartilage dissection to avoid injury to the LITA (Figure 2). After median sternotomy, the LITA and saphenous vein graft were dissected using an ultrasonic scalpel. Cardiopulmonary bypass was instituted by femoral artery and vein cannulation. The heart was then arrested, and myocardial protection was achieved by retrograde cold blood cardioplegia and mild hypothermia with the LITA graft clamped. The pathologic findings from the explanted valve were the presence of pannus adherent to the inflow and outflow portion. Partial tears of the noncoronary cusps from the stent post between the right and noncoronary cusps were observed. After resection of the pannus formation around the annulus, reoperative AVR using a new bioprosthetic valve (INSPIRIS RESILIA aortic valve, 21 mm; Edwards Lifesciences) was performed. He was successfully weaned from cardiopulmonary bypass with inotropic agents.

## 4 OUTCOME AND FOLLOW‐UP

Although antibiotic treatment was required for pneumonia, the patient recovered sufficiently and started rehabilitation 1 week postoperatively. Transthoracic echocardiography 2 weeks after the surgery showed that the mean transaortic gradient was 12 mm Hg without a paravalvular leak; however, the peak velocity was slightly elevated at 2.6 m/s. He was discharged 1 month after surgery and was followed up closely as an outpatient for 1 year.

## 5 DISCUSSION

This report presents a case in which sudden onset structural valve deterioration requiring emergent surgery occurred. Reoperative AVR and preservation of patent bypass grafts were successfully achieved via a modified median sternotomy.

Several reports have shown lower than predicted surgical mortality for transcatheter aortic valve implantation within degenerated aortic surgical bioprostheses. However, there was a higher than average residual mean gradient and ratio of severe patient‐prosthesis mismatch in the transcatheter aortic valve implantation group than in the reoperative AVR group.^2^, ^3^ Although severe patient‐prosthesis mismatch was not predictive of increased early or midterm mortality, the effect on long‐term outcomes remained unclear. Therefore, in addition to patients with a risk of migration of torn cusps as seen in this case, we consider patients with a significant paravalvular leak or severe patient‐prosthesis mismatch to the implanted bioprosthesis as possible candidates for reoperative AVR at this point.

Reoperative AVR achieved acceptable long‐term outcomes, a lower incidence of patient‐prosthesis mismatch, and a lower mean postoperative aortic valve gradient.^1^, ^4^ However, reoperative AVR resulted more frequently in a valve diameter reduction than the size of the existing stentless valve. Excellent hemodynamics were not achieved unless the new prosthesis was identical to or larger than the previous stentless xenograft.^5^ Although root dilatation adds further complexity to reoperative AVR, it is likely to be an essential technique from a hemodynamic standpoint if a patient‐prosthesis mismatch is observed with a previously implanted bioprosthesis.

In cases with coronary grafts traversing the lower sternum, a right minithoracotomy with the LITA no dissection technique would be helpful to avoid injury.^6^ Although this surgical strategy could have been performed in the present case, we chose the most familiar surgical strategy to avoid saphenous vein graft injury in case root dilatation was required.

We successfully managed acute severe congestive heart failure due to structural valve deterioration with reoperative AVR through a modified median sternotomy. Current evidence suggests that the choice between valve‐in‐valve transcatheter aortic valve implantation and reoperative AVR should be based on multiple factors. The surgical strategy for reoperative AVR after CABG should also be tailored to each individual case considering the possibility of root dilatation and the location of bypass grafts.

## CONFLICT OF INTEREST

None declared.

## AUTHOR CONTRIBUTIONS

DK: wrote the manuscript. HW: participated in the surgery and supervised the writing of the manuscript. MO: participated in the surgery. HY: participated in the surgery. All authors read and approved the final manuscript.
